# Active MMP-8 (aMMP-8) as a Grading and Staging Biomarker in the Periodontitis Classification

**DOI:** 10.3390/diagnostics10020061

**Published:** 2020-01-22

**Authors:** Timo Sorsa, Saeed Alassiri, Andreas Grigoriadis, Ismo T. Räisänen, Pirjo Pärnänen, Solomon O. Nwhator, Dirk-Rolf Gieselmann, Dimitra Sakellari

**Affiliations:** 1 Department of Oral and Maxillofacial Diseases, Head and Neck Center, University of Helsinki and Helsinki University Hospital, P.O. Box 63 (Haartmaninkatu 8) FI-00014, 00100 Helsinki, Finland; 2 Division of Periodontology, Department of Dental Medicine, Karolinska Institutet, SE-171 77 Stockholm, Sweden; 3 Department of Preventive Dentistry, Periodontology and Implant Biology, Dental School, Aristotle University of Thessaloniki, 54124 Thessaloniki, Greece; 4 424 General Army Hospital, 54124 Thessaloniki, Greece; 5 Department of Preventive & Community Dentistry, Faculty of Dentistry, College of Health Sciences, Obafemi Awolowo University, Ile-Ife 220104, Osun State, Nigeria; 6 Institute for Molecular Diagnostics (IMOD), Bonner Str. 84, 42697 Solingen, Germany

**Keywords:** periodontitis, diagnosis, matrix metalloproteinase 8, biomarkers, molecular diagnostics, periodontal diseases, point-of-care testing

## Abstract

The aim of this study was to investigate the utility of incorporating active matrix metalloproteinase-8 (aMMP-8) as a biomarker into the new periodontitis classification system (stage/grade) presented in 2018. This study included 150 Greek adults aged 25–78, of whom 74 were men and 76 women. Participants were tested with an aMMP-8 point-of-care mouthrinse test, after which a full-mouth clinical examination was performed to assess their periodontal and oral health. The aMMP-8 levels in mouthrinse were significantly lower among healthy patients compared with patients in more severe periodontitis stages and grades (Kruskal–Wallis test and Dunn–Bonferroni test for pairwise post-hoc comparisons; *p* < 0.01 and *p* < 0.05, respectively). Furthermore, aMMP-8 levels were less correlated with plaque levels than bleeding on probing (BOP) (Spearman’s rho = 0.269, *p* < 0.001; Spearman’s rho = 0.586, *p* < 0.001); respectively). Thus, aMMP-8 was more robust to the confounding effects of oral hygiene than traditional periodontal parameter bleeding on probing. The aMMP-8 point-of-care mouthrinse test can be utilized as an adjunctive and preventive diagnostic tool to identify periodontal disease, classified by stage and grade, and ongoing periodontal breakdown chairside in clinical practice in only 5 min. Overall, integrating aMMP-8 into the new periodontitis classification system seems beneficial.

## 1. Main text

A new revised periodontitis case definition system was introduced in 2018 to define periodontitis more appropriately by its stage (the severity and extent of periodontal tissue destruction and complexity of management), and grade (the future risk of progression) [1]. One of its main goals is to provide dentists a framework for individualized identification, treatment and prevention of periodontitis in patients. In addition, it provides a necessary and very much needed framework for the introduction of diagnostic and prognostic biomarkers to extend and improve the information provided by the standard clinical measures. 

As Tonetti et al. [1] note, there are some limitations regarding reliance on bleeding on probing (BOP) and other traditional measures in periodontitis diagnostics. For example, some of them are very much influenced by the level of training and experience of operator (i.e., periodontal probing) [1]. Furthermore, the clinical measurements provide mainly retrospective evidence of the severity and extent of periodontal breakdown but are limited methods to assess and predict the risk for on-going and future periodontal breakdown [2]. That stems from the nature of periodontitis to progress episodically, as the quiescent and active periods alternate [2]. Periodontal breakdown does not always follow such geometric or arithmetic calculations or linear patterns that are accurately measurable by radiographic bone loss and/or clinical attachment loss (CAL).

The new periodontitis classification system includes grading parameters to assess the future risk of periodontitis progression. Although they are associated with periodontitis and increase the likelihood of future periodontal breakdown, they cannot be considered to be reliable enough to indicate when periodontitis is in its active phase. This applies to even the grade modifiers smoking and diabetes that are well-known risk factors for periodontitis [3,4,5]. The current grading parameters are mainly able to predict that it is likely that the periodontal breakdown occurs in the future, but not the exact time when it is occurring. In this regard, the future inclusion of more robust biomarkers to the classification system is still anticipated in order to identify patients in active periods of periodontitis (= ongoing periodontal breakdown) and to be able to monitor disease progression and treatment responses more accurately [1].

Oral fluids have been studied intensively to find suitable biomarkers from saliva and gingival crevicular fluid (GCF) for the diagnostics of periodontitis. One of the most studied biomarkers is matrix metalloproteinase-8 (MMP-8). Several studies (for example Lee et al. [6], Romanelli et al. [7], Kiili et al. [8], Sorsa et al. [9], and Alassiri et al. [10]) have demonstrated how elevated levels of active matrix metalloproteinase-8 (aMMP-8), but not total or latent MMP-8, differentiate periodontitis from gingivitis, and precede periodontal attachment loss. Furthermore, active periodontal tissue destruction in progress (i.e., the active phase of periodontal disease) can be identified non-invasively in oral fluids as a pathological elevation of aMMP-8 levels [11,12,13]. Thus, it is no surprise that the aMMP-8 chairside/point-of-care (PoC) oral fluid tests have continuously been successful in identifying active periodontal tissue destruction and active periodontal disease (periodontitis, peri-implantitis, subclinical periodontitis) among adults and adolescents of different ethnic populations [10,12,14,15,16,17,18,19,20,21,22,23,24,25,26]. Similarly, previous longitudinal studies have shown that aMMP-8 assays with the same antibody can be used for predicting the progression of periodontitis and attachment loss, and monitoring treatment of periodontitis during the maintenance phase [27,28,29,30,31,32,33,34].

Many other potential biomarkers to detect periodontitis and related systemic diseases have been investigated as well [35,36,37,38,39,40,41,42,43]; these include, along with others, asymmetric dimethylarginine (ADMA), C-reactive protein, interferon gamma (IFN-γ), interleukin-6 (IL-6), macrophage inflammatory protein-1α (MIP-1α), PMN elastase, vitamin C and also antioxidants [35,36,37,38,39,40,41,42,43]. For example, vitamin C is linked to oxidative MMP-activation cascade by its recorded ability to prevent the oxidative activation of proMMP-8 [44]. According to a recent systematic review, aMMP-8/MMP-8 is currently the most accurate diagnostic biomarker in GCF for periodontitis in systemically healthy patients [35]. In saliva, aMMP-8/MMP-8 is also among the best five biomarkers, and the combination of aMMP-8/MMP-8 and IL-6 seems the most promising salivary diagnostic marker for periodontitis [36,37].

In this short communication, the aim is to study the usefulness of aMMP-8 as the stage and grade biomarker for the new periodontitis classification system [1]. Our hypothesis is that aMMP-8 levels among 150 Greek adults, measured by a quantitative aMMP-8 point-of-care (PoC)/chairside mouthrinse test, are positively associated with the severity of periodontal disease (stage) and possible risk for disease progression (grade).

**Figure 1:**
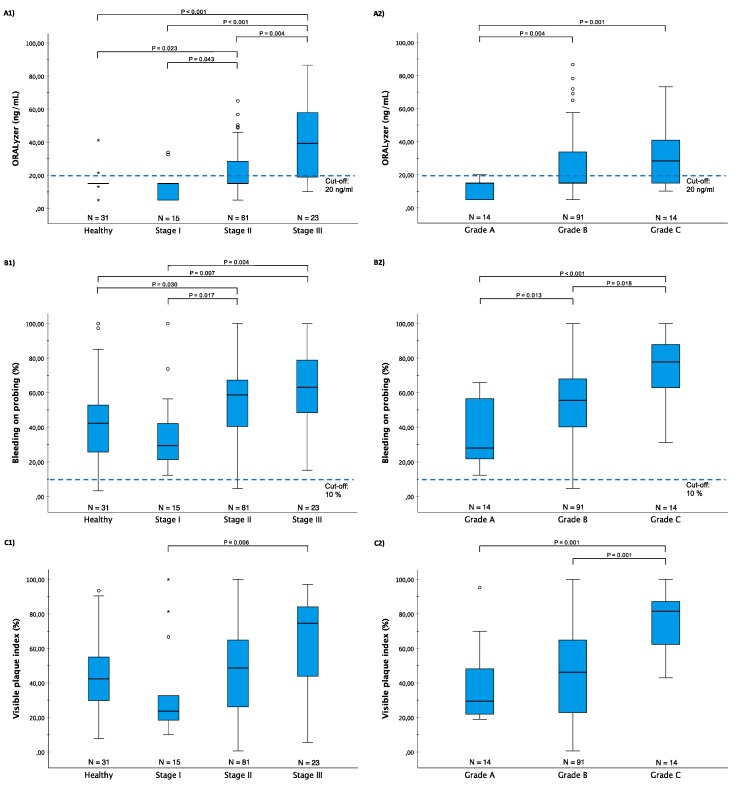
(**A**) ORALyzer (ng/mL), (**B**) bleeding on probing (BOP) (%) and (**C**) visual plaque index (VPI) (%) vs. (1) periodontitis stages and (2) periodontitis grades (N = 150) described by Tonetti et al. [1]. Kruskal–Wallis test was significant (*p* < 0.01) for all the three variables in both cases. All significant (*p* < 0.05) pairwise post hoc comparisons (Dunn–Bonferroni test) are marked in the plots. The box-and-whiskers plots illustrate the median, quartiles, and extreme values. Previously validated cut-off of 20 ng/mL for aMMP-8 assays [10] and cut-off of 10% for BOP [1,49] marked in the figure.

Figure 1 demonstrates the aMMP-8 levels, as well as bleeding on probing (BOP), and visual plaque index (VPI) levels among 150 Greek patients classified by the new classification system [1]. The participants were selected among the patients of the Periodontology University Clinic, Thessaloniki, Greece, as described earlier [26]. They had no underlying diseases and no medications (antibiotics, anti-inflammatory drugs etc.) were in use for the last 3 months before their examination (more information about patient characteristics in Table 1). Patients’ aMMP-8 levels were determined by the aMMP-8 PoC/chairside mouthrinse test, PerioSafe^®^, in combination with a digital reader, ORALyzer^®^, following the manufacturer’s instructions [10,14,24]. A.G. performed a full-mouth clinical examination for each patient to assess their periodontal and oral health, after which each patient’s aMMP-8 mouthrinse test result was read.

Firstly, aMMP-8 levels in mouthrinse were significantly lower among healthy patients compared with patients in more severe periodontitis stages and grades (Kruskal–Wallis test, *p* < 0.01; Figure 1), as would be expected from an effective diagnostic tool to classify periodontal health and disease [14,15,16,17,18,19,20,21,22,23,24,25,26]. This result is also in agreement with previous studies that have shown that aMMP-8 predicts very well future periodontal breakdown (positive predictive values range between 81.8%–94.7%) [35]. Secondly, BOP and VPI levels were also associated with the periodontitis stage and grade (Kruskal–Wallis test, *p* < 0.01); Figure 1), but these two parameters were also moderately correlated with each other (Spearman’s rho = 0.586, *p* < 0.001). The association between dental plaque levels and BOP levels and their potential confounding is well understood in the literature [45]. Previous studies have also shown that BOP poorly predicts future periodontal breakdown (positive predictive value for repeated incidence of BOP was ≤ 30%), but the continuous absence of BOP correlates much better with periodontal stability [2,45,46,47,48]. For example, Joss et al. [48] suggested a cut-off of 20% for BOP, while in the new classification system a threshold of 10% for BOP is suggested to define periodontal health [49]. However, as Figure 1 shows, despite the positive association between BOP and both stage and grade, the two cut-offs are likely to cause a large number of false positives. This seems to apply to VPI as well (see Figure 1), for example, when considering a good oral hygiene as a prerequisite for periodontal health and using a low cut-off for VPI. It is noteworthy that plaque assessments are not considered as an effective measure of periodontitis [50,51,52]. According to Lang et al. [45], dental plaque determines only 20% of the direct risk of the development of periodontitis. 

Finally, in this sample, the range of BOP and VPI levels were wide among healthy patients compared with the diseased patients (stage I–III and Grades A–C). Regarding periodontitis patients, the same applies to BOP and VPI levels among Grade A patients when compared to Grade B patients (Figure 1). This kind of overlapping in BOP and VPI levels between different categories increases the risk for false positive diagnosis, as well. In contrast, the aMMP-8 levels did not exhibit similar overlapping, as BOP and VPI, between the same groups. Furthermore, aMMP-8 levels seemed much more immune to the confounding effects of dental plaque, as the link between aMMP-8 and VPI was somewhat negligible (Spearman’s rho = 0.269, *p* < 0.001). All in all, aMMP-8 was the preferable method for differentiating periodontal health and disease, because the risk of false positives seemed generally much lower for aMMP-8 than BOP or VPI (see Figure 1). 

**Figure 2:**
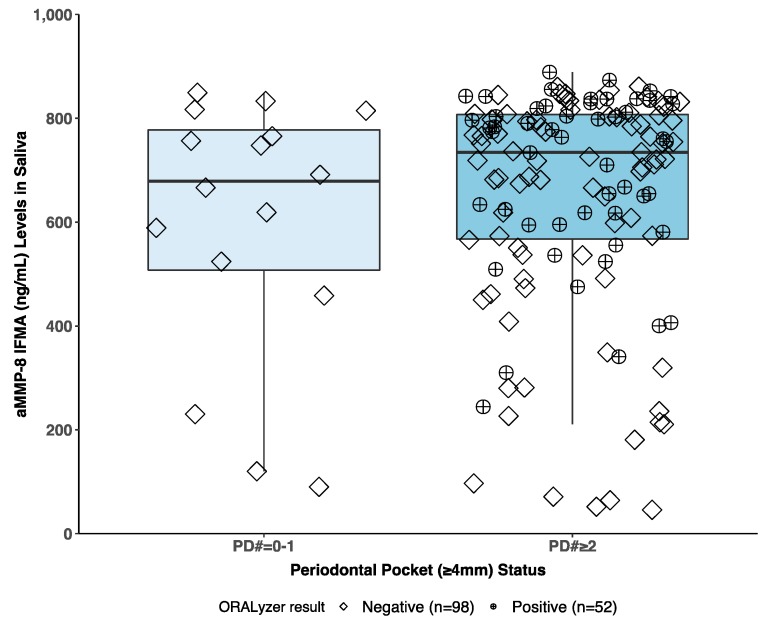
The concentrations of aMMP-8 (ng/mL) in saliva measured by time-resolved immunofluorometric assay (IFMA) according to patient’s periodontal pocket (≥4 mm) status (zero or one [PD# = 0–1] vs. two or more periodontal pockets [PD# ≥ 2]) categorized by the corresponding ORALyzer test result (cut-off 20 ng/mL) measured from oral mouthrinse. A positive and negative ORALyzer results marked as diamonds and circle-plusses, respectively. N = 150 Greek patients.

Our findings indicate that the aMMP-8 mouthrinse PoC/chairside test [10,12,14,15,16,17,18,19,20,21,22,23,24,25,26] can be implemented as the staging and grading biomarker in the new classification system of periodontitis [1]. In this regard, a previous study by Räisänen et al. [21] showed that mouthrinse aMMP-8 measurements are better and more precise in identifying periodontal health and disease among adolescents in comparison to saliva aMMP-8 measurements. The same result was repeated and further extended in this study by comparing the aMMP-8 PoC/chairside mouthrinse test to salivary aMMP-8 time-resolved immunofluorometric assay (IFMA) measurements, both methods utilizing the same antibody, among this sample of Greek adults (see Figure 2) [10,27,29]. The mouthrinse aMMP-8 measurements performed more precisely and gave no false positives (i.e., no positive aMMP-8 mouthrinse test results among healthy patients). In contrast, IFMA measurements of aMMP-8 levels in saliva offered a less definite classification of periodontal health and disease (Figure 2). As Räisänen et al. [21] suggest, mouthrinse aMMP-8 measurements seem the optimal way of measuring the active periodontal breakdown and risk of the future progression of periodontal disease.

The utility of mouthrinse measurements is based on a simple technique to collect and sample gingival crevicular fluid (GCF) [14,21,53], instead of using more laborious filter papers or micropipettes [54]. Moreover, previous studies have also presented a better diagnostic accuracy value of GCF in aMMP-8 measurements in comparison to saliva when classifying periodontal health and disease [35,36,37]. However, it should also be noted that not all commercial kits detect active forms of MMP-8, as the aMMP-8 PoC mouthrinse test and IFMA used in this study do [29]. Instead, some of them detect the total MMP-8, which is not able to detect periodontal breakdown or progression of periodontitis [55,56,57]. This fact may explain the variation in aMMP-8 and MMP-8 results in the literature, in addition to the type of oral fluid used in the testing.

Based on this evidence, therefore, we strongly support the call of Tonetti et al. [1] for a paradigm shift to also consider periodontitis in terms of the inflammatory mediators that are immunoassay measures, offering information that traditionally used diagnostic methods are not able to provide as accurately. We propose aMMP-8 as the biomarker and the aMMP-8 PoC/chairside mouthrinse testing to be incorporated into the new periodontal disease classification system by Tonetti et al. [1] (see Table 2: modified from Tonetti et al. [1]), to improve the diagnostic accuracy of periodontal diseases and their progression. The proposed aMMP-8 measurements by an aMMP-8 PoC mouthrinse test with a cut-off of 20 ng/mL allows this risk to be measured early enough, effectively and non-invasively in clinical practice within 5 min without the need to use laborious and time-consuming laboratory equipment [10,12,14,15,16,17,18,19,20,21,22,23,24,25,26]. The ability to identify active periods of periodontitis (=ongoing periodontal breakdown) chairside and in real-time is of great value to dentists trying to optimize patient treatment. It lets them to monitor disease progression, and treatment responses [10,11,12,14,15,16,17,18,19,20,21,22,23,24,25,26,27,28,29,30,31,32,33,34], which should translate to better treatment outcomes as well. After all, periodontitis does have a negative effect not only to periodontium but also to the whole body by increasing the systemic inflammatory burden and the risk for certain systemic diseases like diabetes [2,4,58]. Finally, in addition to periodontitis, several studies have reported elevated aMMP-8 levels among patients with diabetes (type 1 and 2) [59,60,61,62]. In this regard, the recent study by Grigoriadis et al. [26] demonstrated that aMMP-8 PoC mouthrinse testing is also suitable for prediabetes/diabetes screening at the dental office.

## 2. Patents

Timo Sorsa and Dirk-Rolf Gieselmann are inventors of US-patent 10 488 415 B2 and a Japanese patent 2016-554676.

## Figures and Tables

**Table 1:** Patient characteristics (N = 150 Greek adults).

	Healthy	Stage I	Stage II	Stage III	*p*-Value	Grade A	Grade B	Grade C	*p*-Value
Sex (N)					0.003 ^a^				
Women	11	14	39	12		12	47	6	0.038 ^a^
Men	20	1	42	11		2	44	8	
Age mean (SD)	43.32 (12.78)	61.64 (8.10)	54.96 (9.83)	56.00 (9.61)	<0.001 ^b^	60.64 (11.17)	55.03 (9.54)	57.21 (8.85)	0.200 ^b^
Education level (N)					<0.001 ^a^				0.065 ^a^
Elementary	0	1	2	0		0	1	2	
Middle	2	8	41	19		10	48	10	
Post graduate Studies	9	1	4	0		0	5	0	
Technical school	0	0	3	31		0	4	0	
University	20	5	31	3		4	33	2	
Annual dental visit (N)					0.051 ^a^				0.680 ^a^
Yes	19	14	45	13		9	56	7	
No	12	1	36	10		5	35	7	
Smoking (N)					0.088 ^a^				0.290 ^a^
Yes	6	8	26	10		3	34	7	
No	25	7	55	13		11	57	7	
Normal BMI (N)					0.171 ^a^				0.641 ^a^
Yes	9	1	21	9		4	22	5	
No	22	14	60	14		10	69	9	

N: frequency; SD: standard deviation; BMI: body mass index; ^a^ Pearson Chi-squared test (asymptotic, 2-sided). ^b^ Welch *t*-test.

**Table 2:** Grading a periodontitis patient (modified from Tonetti et al. [1]) by active matrix metalloproteinase-8 (aMMP-8) as the main biomarker for active/progressing periodontal diseases.

Grading a Periodontitis Patient by aMMP-8		Grade A: Slow Rate of Progression	Grade B: Moderate Rate of Progression	Grade C: Rapid Rate of Progression
Indicators of active periodontal tissue destruction/bone loss/clinical attachment loss	Mouthrinse, gingival crevicular fluid	No/slow = aMMP-8 level < 20 ng/mL	Moderate = aMMP-8 level ≥ 20 ng/mL	Rapid = aMMP-8 level > 30 ng/mL
